# Cleaning the dose falloff in lung SBRT plan

**DOI:** 10.1002/acm2.13113

**Published:** 2020-12-07

**Authors:** Dharmin Desai, Ganesh Narayanasamy, Milan Bimali, Ivan Cordrey, Hisham Elasmar, Senthamizhchelvan Srinivasan, Ellis Lee Johnson

**Affiliations:** ^1^ Memorial Hospital Chattanooga TN USA; ^2^ Department of Radiation Oncology University of Arkansas for Medical Sciences Little Rock AR USA; ^3^ Department of Biostatistics University of Arkansas for Medical Sciences Little Rock AR USA; ^4^ Department of Radiation Medicine University of Kentucky Lexington KY USA

**Keywords:** dose falloff, dose gradient, dose spill, lung SBRT, normal tissue toxicity

## Abstract

**Purpose:**

To investigate a planning technique that can possibly reduce low‐to‐intermediate dose spillage (measured by R50%, D2cm values) in lung SBRT plans.

**Materials and Methods:**

Dose falloff outside the target was studied retrospectively in 102 SBRT VMAT plans of lung tumor. Plans having R50% and/or D2cm higher than recommended tolerances in RTOG protocols 0813 and 0915 were replanned with new optimization constraints using novel shell structures and novel constraints. Violations in the RTOG R50% value can be rectified with a dose constraint to a novel shell structure (“OptiForR50”). The construction of structure OptiForR50% and the novel optimization criteria translate the RTOG goals for R50% into direct inputs for the optimizer. Violations in the D2cm can be rectified using constraints on a 0.5 cm thick shell structure with inner surface 2cm from the PTV surface. Wilcoxon signed‐rank test was used to compare differences in dose conformity, volume of hot spots, R50%, D2cm of the target in addition to the OAR doses. A two‐sided *P*‐value of 0.05 was used to assess statistical significance.

**Results:**

Among 102 lung SBRT plans with PTV sizes ranging from 5 to 179 cc, 32 plans with violations in R50% or D2cm were reoptimized. The mean reduction in R50% (4.68 vs 3.89) and D2cm (56.49 vs 52.51) was statistically significant both having *P* < 0.01. Target conformity index, volume of 105% isodose contour outside PTV, normal lung V20, and mean dose to heart and aorta were significantly lowered with *P* < 0.05.

**Conclusion:**

The novel planning methodology using multiple shells including the novel OptiForR50 shell with precisely calculated dimensions and optimizer constraints lead to significantly lower values of R50% and D2cm and lower dose spillage in lung SBRT plans. All plans were successfully brought into the zone of no RTOG violations.

## 1 INTRODUCTION

For medically inoperable nonsmall cell lung cancer (NSCLC) stages I and II, stereotactic body radiotherapy (SBRT) has been shown to be as effective and safe as surgery in several clinical trials.^1^, ^2^, ^3^ Early‐stage NSCLC patients who underwent SBRT were reported to have had a 3‐yr tumor control rate of up to 98% and a low risk of radiation‐related toxicity.^4^, ^5^, ^6^

American association of physicists in medicine’s (AAPM) task group report #101 states that steeper dose falloff outside the target helps minimize normal tissue toxicity and spare nearby organs at risk (OAR).^7^ However, stereotactic radiotherapy faces multiple challenges from normal tissue toxicity,^8^ tumor motion,^9^ interplay between tumor motion and multileaf collimator (MLC)^10^ among others. With recent technological advances, volumetric modulated arc therapy (VMAT) presents highly conformal dose distribution and fast delivery times. Noncoplanar VMAT plan,^11^ prescribing to lower isodose lines^12^ was some of the techniques reported in a SBRT planning review by Giglioli et al.^13^ Radiation therapy oncology group (RTOG) protocols 0813 and 0915 uses R50% (ratio of volume of 50% prescription isodose volume to the volume of PTV), D2cm (maximum dose at 2 cm from PTV in any direction), and V105% (volume outside the PTV receiving at least 105% of prescription dose) to evaluate the dose falloff. RTOG protocols 0813/0915 recommends tolerance values for two action levels — no deviation and minor plan deviation.^14^, ^15^ Values of R50% and D2cm that are less than the lower bound listed ($LB_{R50\%}^{\mathrm{RTOG}}andLB_{D2cm}^{\mathrm{RTOG}},$ respectively) meet the constraints. While values between respective lower bounds ($LB_{R50\%}^{\mathrm{RTOG}}andLB_{D2cm}^{\mathrm{RTOG}}$) and upper bound ($UB_{R50\%}^{\mathrm{RTOG}}andUB_{D2cm}^{\mathrm{RTOG}}$) tolerance are considered a minor violation, and values above upper bounds are judged a major violation. Note that the tolerance values stated in Table 1 in RTOG protocols 0813 and 0915 correspond to centrally and peripherally located lung tumors, respectively. While experienced treatment planners know effective and proven optimization techniques to lower the normal tissue toxicity, some of them can be cumbersome and time‐consuming. In our study, we have identified an effective method to lower OAR toxicity that is validated using RTOG recommended tolerances.

**Table 1:** Suggested optimization parameters and relative penalties for lung SBRT plans.

Structure	Volume (%)	Min Dose (% of Rx)	Max Dose (% of Rx)	Mean Dose (% of Rx)	Penalty (relative number)
PTV	0	–	140	–	200
	100	100	–	–	200
OptiForR50	0		100	–	100
	%Vopti		45	–	200
Shell1	0		100	–	100
	–		–	$D_{\mathrm{av}}^{Shell1}$	200
Shell2	0		76	–	100
Shell3	0		63	–	100
Shell4	0		56	–	100
Shell5	0		$LB_{D2cm}^{\mathrm{RTOG}}$	‐	200

Shell1 is a 5mm expansion outside the PTV. Shell2 – Shell5 are concentric nonoverlapping shells with each being a 5 mm expansion of the previous inner shell. OptiForR50 is a shell expansion of the PTV with the expansion given by M in Eq. (14), and that overlaps Shell1 – Shell5, where %Vopti is given by Eq. (15). $D_{\mathrm{Av}}^{Shell1}$ is given by Eq. (1) and the RTOG limit for D2cm = $LB_{D2cm}^{\mathrm{RTOG}}$.

## 2 MATERIALS AND METHODS

### 2.A Patient population

Retrospective analysis was performed on 102 clinically implemented and delivered RapidArc‐based VMAT lung SBRT plans. The patients were immobilized in the supine position using Body Pro‐Lok™ platform (CIVCO system, Orange City, IA) with arms above their head. Respiration correlated 4D CT scan was obtained on a GE Lightspeed CT scanner (GE Healthcare, Chicago, IL) at 2.5 mm slice spacing. Average intensity projection (AIP) and maximum intensity projection (MIP) images were generated from four‐dimensional computed tomography (4D CT) scan and exported to Eclipse treatment planning system (TPS) version 11 (Varian Medical Systems, Palo Alto, CA). The two image series were coregistered and internal target volume (ITV) was manually segmented on the MIP image series before transferring over to AIP images for dose computation. PTV was generated with a nonuniform 5 mm margin along axial and 10 mm margin in longitudinal plane to accommodate setup errors. Bilateral lungs, spinal cord, esophagus, heart, great vessels, and ribs were some of the OARs contoured on the AIP following the RTOG protocols 0813/0915.

### 2.B Treatment planning

Highly conformal RapidArc‐based VMAT plans were generated with AAA calculation algorithm using 6 and 10 MV photon beams on a TrueBeam linac. VMAT‐based inverse optimization includes variable gantry speed, dose rate, and MLC leaf positions. Dose of 48–50 Gy in four fractions was prescribed to 95% of volume of PTV (D95%). Collimator angle was optimized and partial arcs were used to avoid entrance dose to contralateral lung.

### 2.C Dose falloff in a cohort

Patient plans were evaluated using RTOG conformity index (CI), R50%, D2cm, V105%, OAR dose tolerances including percent volume of normal lung irradiated by 20 Gy isodose cloud (V20). All plans met the CI and OAR dose tolerances as specified in RTOG protocols 0813/0915, although the treatment preceded the clinical implementation of the RTOG protocols. A cohort of 32 patient plans having deviations in R50% or D2cm including 1 with major deviation in R50% was identified. Upon further scrutiny, dose falloff gradient varied systematically from 257 cGy/mm to 42 cGy/mm in the first 5 mm and mean dose falloff of at least 120 cGy/mm was noticed in the first 2 cm outside PTV. As R50% and D2cm are dependent on the maximum dose outside the PTV, the maximum dose in a 5 mm shell outside the PTV was investigated. The 50% isodose cloud (IDC50%) was always within 3 cm from the PTV surface in the transverse direction and 1 cm in the longitudinal direction.

### 2.D Replanning methodology

The aim of the replanning strategy was to meet the RTOG standard for CI, V105%, R50% ($LB_{R50\%}^{\mathrm{RTOG}}$) and D2cm ($LB_{D2cm}^{\mathrm{RTOG}}$). Thus, we devise a set of optimization shells and most importantly optimizer criterion that clearly push the optimizer toward the desired solution. First, create a series of five nested shells each with a 5 mm expansion of the previous shell, and no shell overlaps with any other shell, but they do share borders. These shells are numbered 1–5 with “Shell1” bordering the PTV, and “Shell5” being the outermost shell.

Shell1 is used to force conformity of the high dose region that controls the high dose spill (V105%). Because Shell1 shares its inner border with the PTV surface, the maximum dose (Dmax) constraint on Shell1 is set to the prescription dose (Rx). Furthermore, the mean percent dose of Shell1 ($D_{\mathrm{Av}}^{Shell1}$) constraint is given by the empirical formula derived in a pilot study on over 100 delivered lung SBRT plans:

(1) $$D_{\mathrm{av}}^{\mathrm{Shell}1}=0.08\times V_{\mathrm{PTV}}+75.67$$

Shell2–Shell5 are used to encourage a steep dose gradient with parameters specified in Table 1. Shell2–4 are not necessary as long as Shell5 is properly constructed with its inner surface 2cm from the PTV surface; yet they provide an extra measure of control over the optimization that is helpful.

Shell5 has an inner edge that is 2 cm from the PTV surface. Thus, Shell5 is used to enforce conformance to the D2cm criterion from the RTOG 0915 Table 1 ($LB_{D2cm}^{\mathrm{RTOG}}$). Since the RTOG D2cm criterion is dependent on the PTV volume, these criteria must scale with the PTV volume. This sets a maximum dose optimizer criterion for Shell5 (see Table 1).

To directly control the R50%, we must construct a new optimization shell and translate R50% and the Lower Bound guidelines of RTOG 0915 into appropriate inputs for the optimizer. We call the new shell “OptiForR50.” Its total volume must be constructed such that the shell is large enough that the entire IDC50%shell (defined in Eq. 2) is within the OptiForR50 shell. In region of interest (ROI) algebra terms:

(2) IDC50\%shell=IDC50\%‐PTV

We find that in the Eclipse optimizer the OptiForR50 shell must also be small enough that the IDC50%shell occupies about 20% (1/5) of the OptiForR50 shell volume based on a pilot study on over 100 delivered lung SBRT plans. Thus,

(3) $$\frac{1}{5}=\frac{\mathrm{IDC}50\text{\textbackslash{}\%}\;\mathrm{shell}}{\mathrm{OptiForR}50}$$

Noting that R50% is given by.

(4) $$R50\%=\frac{\mathrm{IDC}50\%}{\mathrm{PTV}}=\frac{\mathrm{IDC}50\%\mathrm{shell}+\mathrm{PTV}}{\mathrm{PTV}}=\frac{\mathrm{IDC}50\%\mathrm{shell}}{\mathrm{PTV}}+1$$

Thus,

(5) $$\mathrm{IDC}50\text{\textbackslash{}\%}\;\mathrm{shell}=\left(R50\text{\textbackslash{}\%}\;‐1\right)\times \mathrm{PTV}$$

The volume of the IDC50%shell that meets the RTOG R50% criterion (${\mathrm{LB}}_{R50\text{\textbackslash{}\%}\;}^{\mathrm{RTOG}}$) is given by:

(6) $$\mathrm{IDC}50\text{\textbackslash{}\%}\;\mathrm{shell}=\left({\mathrm{LB}}_{R50\text{\textbackslash{}\%}\;}^{\mathrm{RTOG}}‐1\right)\times \mathrm{PTV}$$

Substituting Eq. (6) into Eq. (3) with a little algebra yields.

(7) $$\mathrm{OptiForR}50=5\times \left(\left({\mathrm{LB}}_{\mathrm{RTOG}}‐1\right)\times \mathrm{PTV}\right)$$

Now we need to translate this into a volumetric expansion that can be used as an input in the planning system: an expansion by a margin M given by the ROI algebra expression:

(8) $$\mathrm{OptiForR}50=\left(\mathrm{PTV}+M\right)‐\mathrm{PTV}$$

For simplicity, we will work with an equivalent sphere such that:

(9) $$V_{\mathrm{PTV}}=\frac{4\pi }{3}{\left(r_{\mathrm{PTV}}\right)}^{3}$$

Thus, Eq. (8) becomes.

(10) $$V_{\mathrm{OptiForR}50}=\frac{4\pi }{3}{\left(r_{\mathrm{PTV}}+M\right)}^{3}‐\frac{4\pi }{3}{\left(r_{\mathrm{PTV}}\right)}^{3}$$

And Eq. (7) becomes.

(11) $$V_{\mathrm{OptiForR}50}=5\times \left({\mathrm{LB}}_{R50\text{\textbackslash{}\%}\;}^{\mathrm{RTOG}}‐1\right)\times \frac{4\pi }{3}{\left(r_{\mathrm{PTV}}\right)}^{3}$$

Clearly, we can combine Eqs. (10) and (11) and divide both sides by 4 π/3 to get:

(12) $$5\times \left({\mathrm{LB}}_{R50\text{\textbackslash{}\%}\;}^{\mathrm{RTOG}}‐1\right)\times {\left(r_{\mathrm{PTV}}\right)}^{3}={\left(r_{\mathrm{PTV}}+M\right)}^{3}‐{\left(r_{\mathrm{PTV}}\right)}^{3}$$

We seek the value of M. Simple algebraic manipulation yields:

(13) $$M=r_{\mathrm{PTV}}\times \left\{{\left(5\times {\mathrm{LB}}_{R50\text{\textbackslash{}\%}\;}^{\mathrm{RTOG}}‐4\right)}^{1/3}‐1\right\}$$

Plugging (9) in (13) gives us.

(14) $$M={\left(\frac{3}{4\pi }V_{\mathrm{PTV}}\right)}^{1/3}\times \left\{{\left(5\times {\mathrm{LB}}_{R50\text{\textbackslash{}\%}\;}^{\mathrm{RTOG}}‐4\right)}^{1/3}‐1\right\}$$

This yields a shell that overlaps Shell1–Shell5 and will be used in the optimizer to limit R50%.

Next, we need to translate the ${\mathrm{LB}}_{R50\text{\textbackslash{}\%}\;}^{\mathrm{RTOG}}$ into a set of criteria that can be used as inputs to the optimizer. We calculate the percent of the volume of the OptiR50 shell that must get less than 50% of the prescription dose to satisfy the RTOG R50% criterion, %Vopti.

(15) $$\%\mathrm{Vopti}=100\times \frac{\left({\mathrm{LB}}_{R50\%}^{\mathrm{RTOG}}‐1\right)\times V_{\mathrm{PTV}}}{V_{\mathrm{OptiForR}50}}$$

This gives a percent volume (%Vopti) target goal value needed to achieve the lower bound RTOG constraint (${\mathrm{LB}}_{R50\text{\textbackslash{}\%}\;}^{\mathrm{RTOG}})$. If OptiForR50 is constructed according to Eq. (8) based on margin in Eq. (14) observing dosimetric criteria in Eq. (15), the value of %Vopti ≈ 20%.

It is important to ask the optimizer for a little less than 50% of the prescription dose to ensure the optimizer is forced to seek the best possible plan. Yet asking for something that is well beyond the physically achievable result can lead to significant time wasted in the optimization chasing an impossible result. Empirically we have determined that 45% of the prescription dose is a good target. This value is designated in Table 1 along with the other parameters and the relative penalties. Note the values given in Table 1 are derived empirically and may need alteration to meet your clinical needs.

We find the specific criteria stated above work well in the Eclipse optimizer. Below we give a more general expression for M where the volume target is not based on 20% (1/5) but on a generalized percent target P% (P/100).

(16) $$M={\left(\frac{3}{4\pi }V_{\mathrm{PTV}}\right)}^{1/3}\times \left\{{\left[1+\left(\frac{100}{P}\right)\times \left(LB_{R50\%}^{\mathrm{RTOG}}‐1\right)\right]}^{\text{1}\;/\text{3}}‐1\right\}$$

where *P* = *the desired percentage of OptiForR50 occupied by the IDC50\% shell*.

### 2.F Dose statistics

The revised plans were studied for dosimetric acceptability per RTOG protocols 0813/0915 based on CI, R50%, D2cm, V105%, and OAR dose constraints. Bivariate scatterplots with fitted splines were used to explore trend between dosimetric measurements and PTV size. The differences in distribution across the two treatment plans were assessed using Wilcoxon signed‐rank test. A two‐sided *P* < 0.05 was considered statistically significant.

## 3 RESULTS

### 3.A Replan

**Fig. 1:**
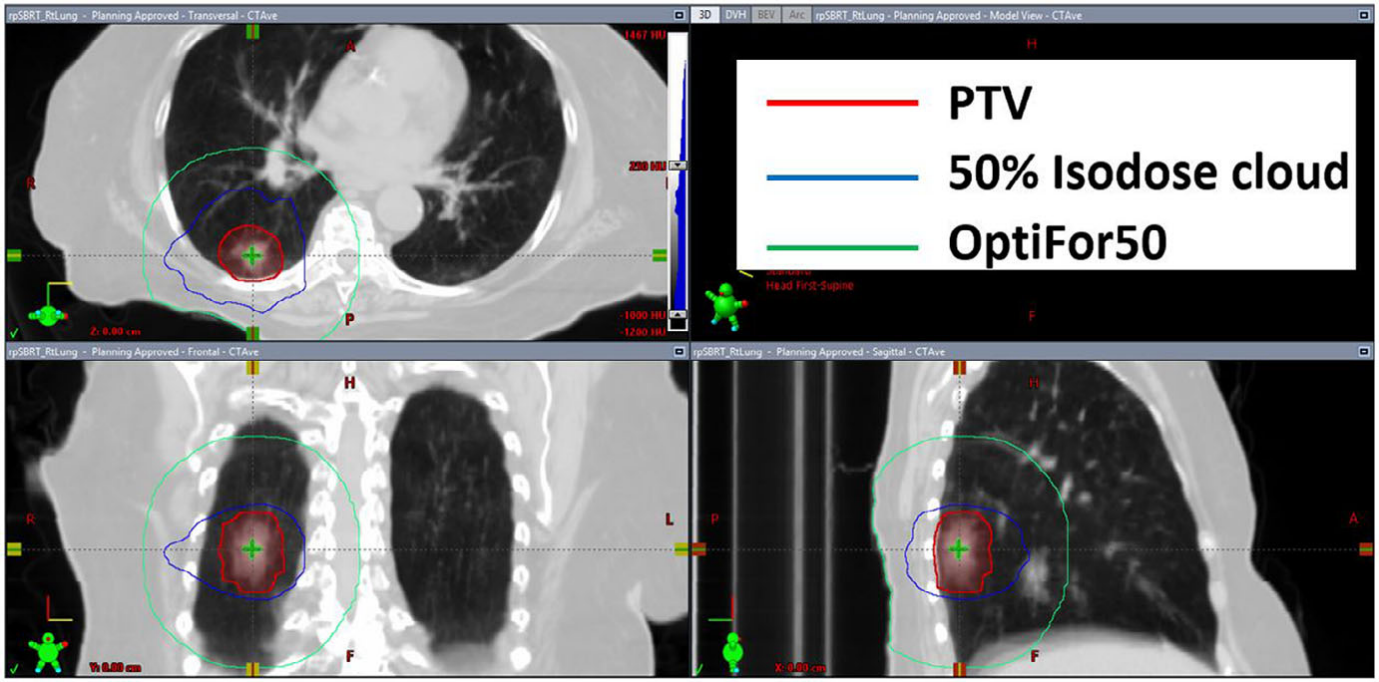
Three‐dimensional orthogonal views of a representative patient plan showing the PTV (=46.1cc), 50% isodose curve (202.8 cc), and OptiForR50 contours.

**Fig. 2:**
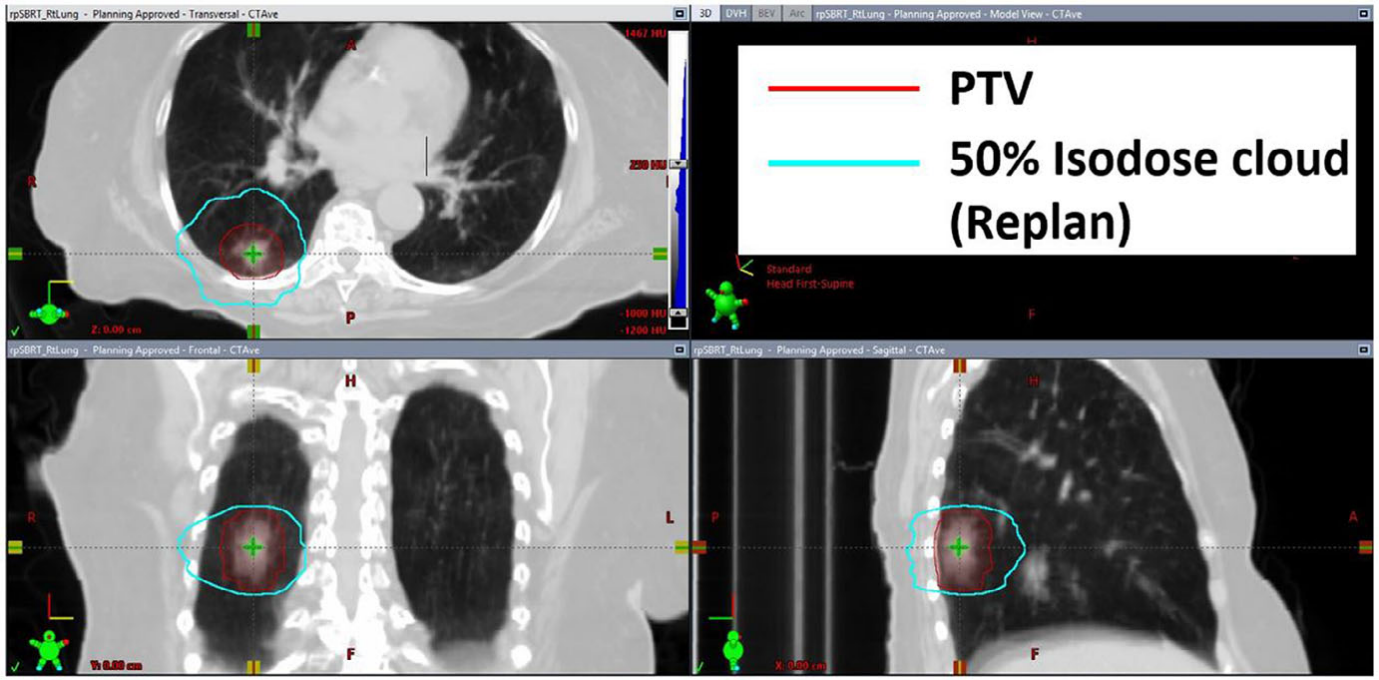
Views of the corresponding replan (as shown in Fig. 1) with a 50% isodose curve (179.4 cc) that is 11.6% smaller in volume.

In the selected cohort, PTV volumes ranged from 5.3 to 179.4 cc (mean ± standard deviation (SD) = 42.2 ± 41.2 cc) with tumor(s) located in all five lobes of either lung. The frequency distribution of PTV volumes is five tumors in range of 5–10 cc, 13 in 10–20 cc, 5 in 25–50 cc, 7 in 65–100 cc, and 2 in 100–200 cc. All the replanned cases consisted of two coplanar arcs for delivery on a Varian Truebeam linac. Beams were tailored to avoid entrance through contralateral lung, although exiting through contralateral lung was unavoidable. Shown in Fig. 1 is a representative plan that did not meet the RTOG criterion for R50%. Shown in 3D orthogonal views are contours of the PTV (in brick red) surrounded by IDC50% (in orange) encompassed within OptiForR50 (in green). Values of both R50% (4.4) and D2cm (66.0%) in the plan in Fig. 1 were higher than the respective $LB_{R50\%}^{\mathrm{RTOG}}$ values of 4.1 and 61.1% (interpolated from data in Table 1 in RTOG protocols 0813/0915). The corresponding replan based on the methodology mentioned earlier is shown in Fig. 2 that includes contours of PTV and the updated but smaller IDC50% (in cyan). Replanning lowered their values of R50% to 3.89 and D2cm to 60.5% thereby making the plan acceptable.

### 3.B Tumor statistics

**Fig. 3:**
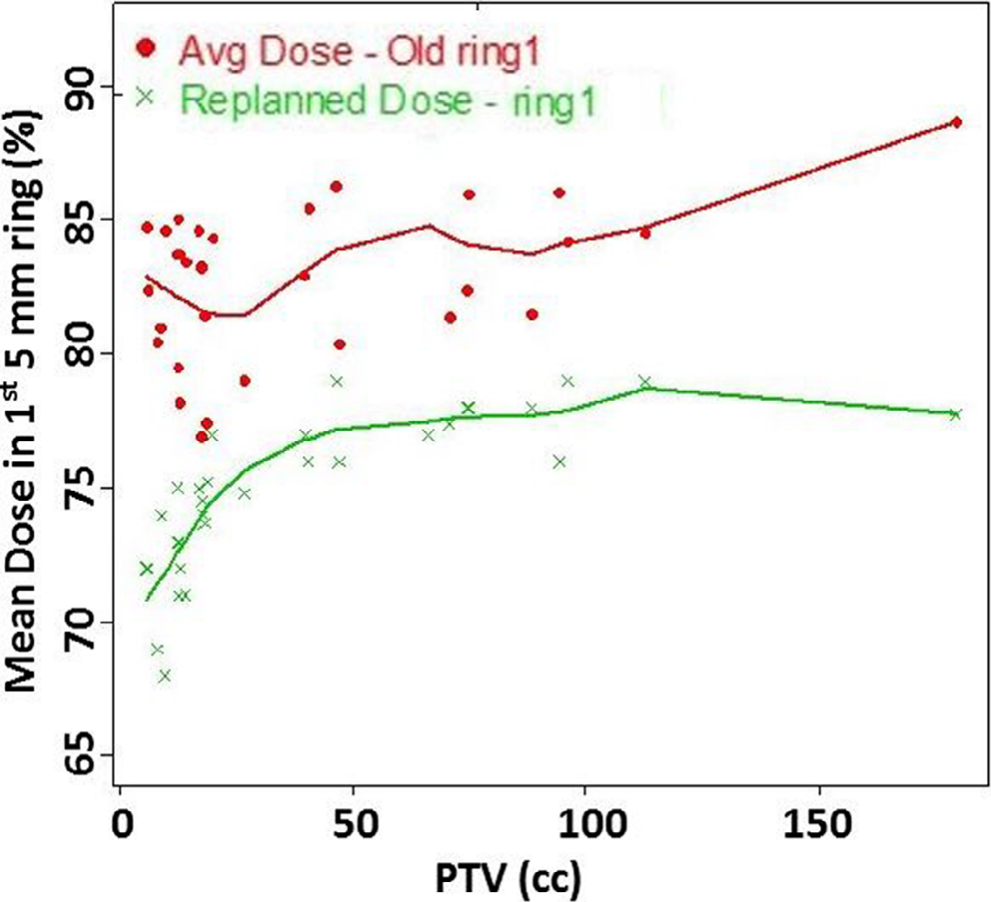
Mean percent dose in the first 5 mm ring structure was lowered by 8% with replanning.

**Fig. 4:**
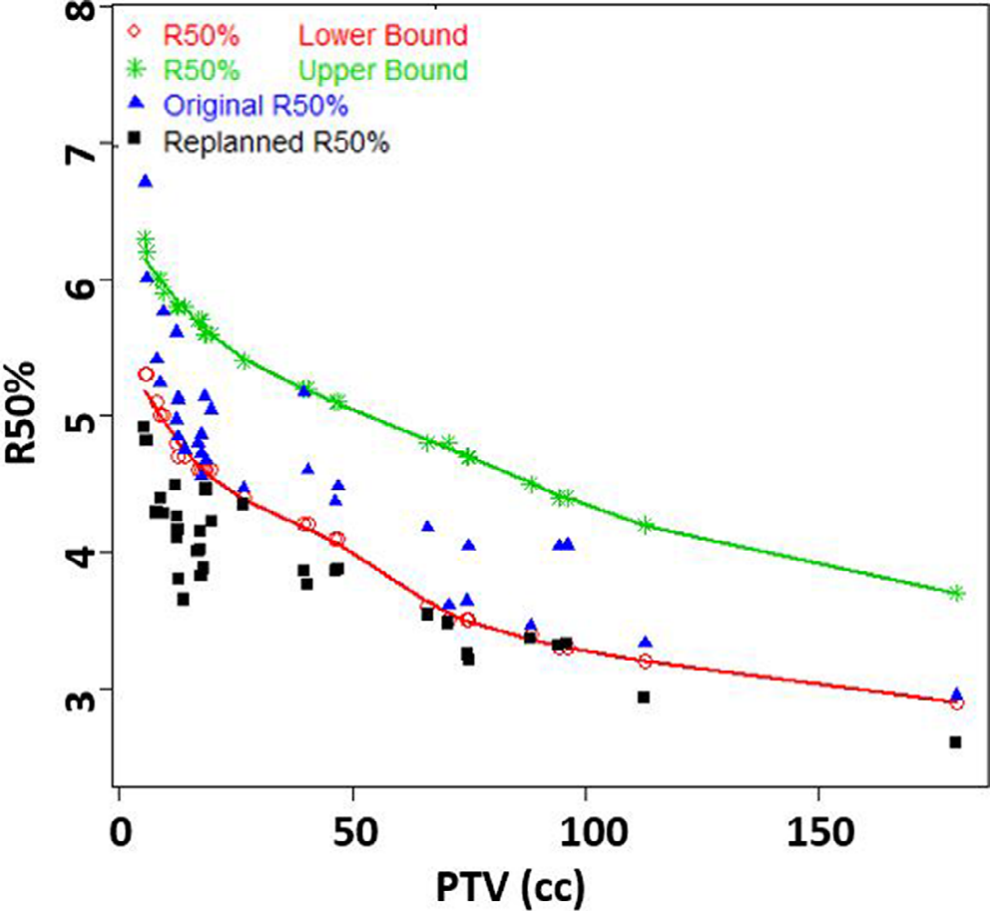
Mean R50% values were lowered from 4.7 to 3.9 with our replanning strategy.

**Fig. 5:**
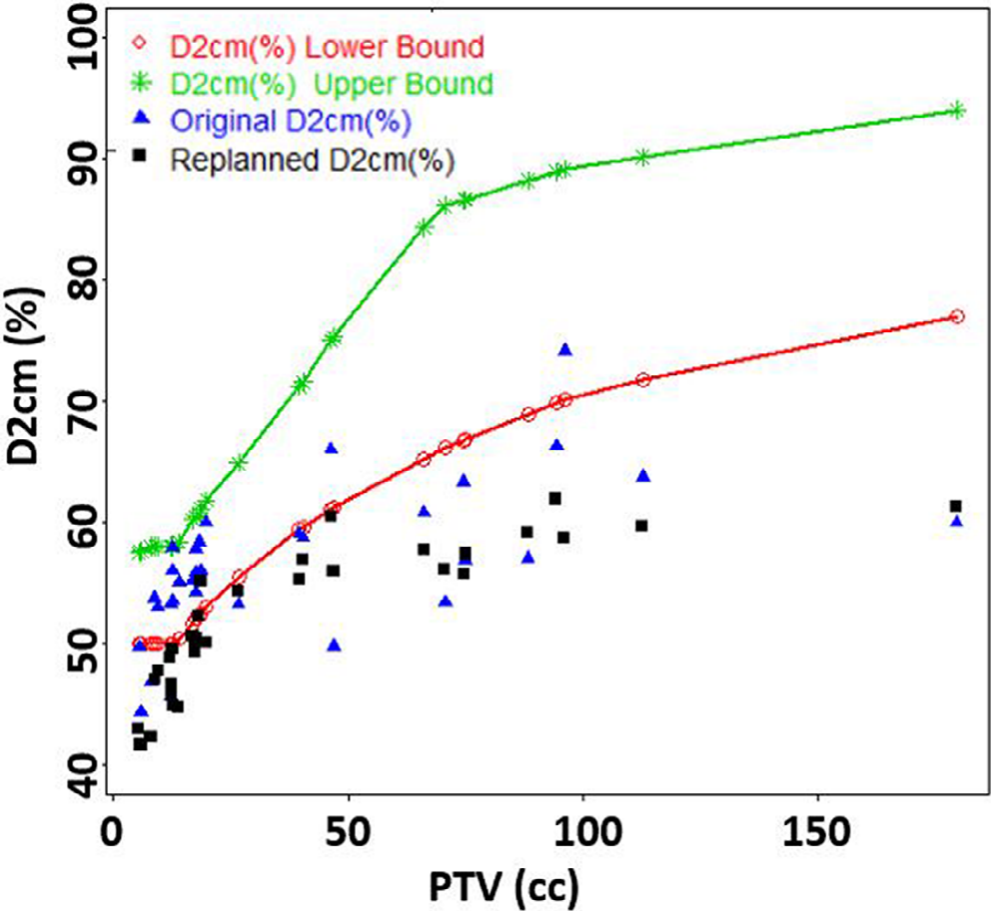
Mean values of D2cm (%) decreased from 56.5% to 52.2% with replanning.

Upon in‐depth evaluation of the mean percent dose in the innermost 5 mm shell (Shell1) outside PTV, a majority of plans were found to have a mean dose above 80% of Rx. The choice of 5 mm shells arose from balancing spatial resolution with data fluctuations, as reported earlier.^16^ The mean ± SD of average %dose in Shell1 of 83 ± 2.9 % was lowered to 75 ± 2.9 % with replanning. With replanning, CI improved from 1.07 ± 0.07 to 1.00 ± 0.01. D2cm (as a percent of Rx) for the original plans was 56.5 ± 6.2%, was lowered to 52 ± 6% upon replanning. Furthermore, R50% of 4.7 ± 0.8% was lowered to 3.9 ± 0.5% with replanning. V105% decreased drastically from 3.57 ± 2.5 to 0.2 ± 0.32. The distribution of all these 5 metrics were found to be significantly different based on Wilcoxon signed‐rank test (*P* < 0.01). The mean % dose in Shell1, R50%, and D2cm (%) on the original and revised plans were plotted against PTV (cc) in Figs. 3, 4, 5, respectively. With replanning, the mean number of monitor units (MUs) were found to be higher by 10.2% from 4046 ± 1060 to 4459 ± 1441 (*P* < 0.01). The differences in maximum dose to the target were not statistically significant (*P* = 0.50). Statistics of the plan comparison with CI, R50%, D2cm, mean dose in Shell1, V105%, MUs, and maximum target dose are summarized in Table 2. Values of R50% and D2cm were lowered in all plans with the replanning methodology making them acceptable per RTOG protocols 0813/0915.

**Table 2:** Comparison of plan evaluation parameters for the original plan and replan on all 32 lung SBRT patients.

Metric	Original plan Mean (SD)	Replan Mean (SD)	*P*‐value
CI	1.07 (0.1)	1.00 (0.0)	<0.01
R50%	4.68 (0.8)	3.89 (0.5)	< 0.01
D2cm (% Rx)	56.49 (6.2)	52.21 (6.0)	< 0.01
Shell1 Average %Dose	82.97 (2.9)	74.89 (2.9)	< 0.01
V105% (%)	3.57 (2.5)	0.21 (0.3)	<0.01
MUs	4046.44 (1059.9)	4459.47 (1441.5)	< 0.01
Max target dose (%)	120.16 (7.4)	119.18 (5.8)	0.50

### 3.C Dose to OARs

Because of our replanning methodology, the impact on OAR doses was found to be significant on heart, aorta, and normal lung as presented in Table 3. The reduced V20 (%) to normal lung tissue from 3.9 ± 3.1 to 3.4 ± 2.7 was significant (*P* < 0.01).The mean ± SD doses to heart and aorta decreased significantly from 1522 ± 1661 to 1485 ± 1726 cGy, and 1705 ± 1023 to 1530 ± 1395 cGy, respectively (*P* < 0.05). The doses to spinal cord, esophagus, skin, bronchus, and other OARs met the RTOG protocols 0813/0915, however, the differences did not attain statistical significance (*P* > 0.05).

**Table 3:** Average values of absolute dose differences between the original plans and replan for OARs on 32 lung SBRT patients.

OAR and Metric	Original plan Mean dose (SD)	Replan Mean dose (SD)	*P*‐value
Normal lung V20 (%)	3.94 (3.1)	3.43 (2.7)	< 0.01
Heart D_av_(cGy)	1521.78 (1660.8)	1485.41 (1726.2)	0.03
Aorta D_av_ (cGy)	1704.75 (1022.8)	1529.91 (751.0)	0.04
Bronchus D_av_(cGy)	1209.5 (1551.4)	1198.25 (1549.0)	0.14
Esophagus D_av_(cGy)	1274.26 (927.9)	1204 (786.9)	0.21
Spinal Cord D_av_(cGy)	926.31 (389.7)	965.78 (428.4)	0.54
Skin D_av_(cGy)	1870.44 (629.9)	1857.44 (671.5)	0.67

## 4 DISCUSSION

In this retrospective study, we have presented our initial experiences of an effective replanning methodology for limiting normal tissue toxicity in the SBRT of a lung tumor following the guidelines of RTOG protocols 0813/0915. None of the replans were used for patient treatment but a sample was measured for standard quality assurance to be sure the plans are deliverable.

An optimization structure, OptiForR50 is created according to the specifications that are detailed in the methods. The corresponding set of optimization parameters is encapsulated in Table 1. Figures 1 and 2 display the delivered plan and replan with OptiForR50 structure along with corresponding 50% isodose contours. A novel Eq. (14), gives a definitive expansion margin on the PTV to achieve the desired OptiForR50 shell. Equation (8) gives a definitive formula to calculate the needed volumetric constraint for the optimizer to push the optimizer toward the desired solution (a plan that conforms to the RTOG R50% standard). Both Eqs. (8) and (14) automatically scale to the RTOG “Lower Bound” ($LB_{R50\text{\textbackslash{}\%}\;}^{\mathrm{RTOG}}$) in R50% for an acceptable plan per RTOG protocols 0813/0915. Other optimization shells assist with creating other desired final plan characteristics: V105%, D2cm, CI.

Other optimization shells are also employed that are a more conventional set of 5mm thick nested shells. It was noticeable that the mean dose to first 5 mm shell structure (Shell1) outside the PTV was higher than 80% on the majority of plans that had minor deviations in R50% and D2cm (plans that did not pass the RTOG criteria) as shown in Fig. 3. With replanning, according to the methodology presented here, the Shell1 mean dose was reduced by 8% while R50% and D2cm (%) were lowered by 0.8 and 4.3%, respectively, thereby making all plans acceptable. Dose conformity and volume of hot spots improved significantly with this replanning methodology as stated in Table 1. However, a limitation of the study was the time for optimizing the delivered and the reoptimized plans were not tracked that may have led to differences in the MUs with replanning. Although the number of MUs increased by 10% with replanning, the maximum dose to PTVs remain practically unchanged. Other replanning strategies are available in literature. A recent publication by Hoffman et al. utilizes a shell structure of width numerically equal to the Gradient Measure (in cm) to restrict dose to the PTV.^17^ This metric based on quantile regression analysis provides a prospective method to lower RX% (where X ranges between 10% and 90%) that is simpler to use than knowledge‐based plan. However, it does not account for variations in PTV shape, PTV separation from OARs etc.

Regarding OAR doses, mean doses to heart, aorta, and V20 of normal lungs were significantly reduced in replans as tabulated in Table 3. Per RTOG protocols 0813/0915 recommendations for lung SBRT plans, V20 < 10–15%. Although the reported toxicities of lung SBRT have been a few and limited, the dose constraints used in treatment planning are based on very limited clinical data as seen in QUANTEC article on lung tumors.^18^ Yamashita et al stated that radiation pneumonitis is one of the most common toxicities after lung SBRT with rates reported from 9% to 28%.^19^, ^20^ In a review article on complications from lung SBRT, radiation pneumonitis, vascular issues, and neuropathy were reported.^21^ Besides mean lung dose (MLD) and volume of 5 Gy isodose cloud, V20 is a well‐studied parameter that correlates with radiation pneumonitis.^22^, ^23^ Perhaps Dong’s 4π planning technique improves tumor coverage and spares critical organs but involves longer treatment delivery with numerous noncoplanar beam suffering from possible collision issues and intrafraction patient motion.^24^ In comparison, our replanning methodology does not involve additional noncoplanar beams and should not add to delivery time. In addition, our approach can be adapted to any target site (lung, cranial, liver, spine, etc) and is scalable to isodose clouds other than 50%. In this retrospective investigation on replanning, the lung SBRT plans were chosen due to the potential impact in lowering normal lung and other OAR doses significantly. One of the constraints of our treatment delivery is that jaw tracking feature was not enabled and enabling this could lead to additional reduction in normal tissue toxicity.

The toxicities associated with lung SBRT plans that include fatigue, chest wall pain, cough, and skin erythema are predominantly mild and short lived. Serious toxicities such as pneumonitis^25^ and fatal esophageal ulceration^26^ had been reported. Sometimes, it is difficult to establish the relative importance of total dose, fractionation, treatment technique (including treatment margin) to toxicity.^27^ Although the dose falloff as a function of distance from PTV has been reported,^16^ there is a lack of clinical data that correlates metrics R50% and D2cm with toxicity scores. In the future, clinical follow‐up is necessary to determine the tumor control rates and study the correlation of lung toxicity with the studied dosimetric parameters.

The optimization methodology described translates the RTOG criterion into a carefully constructed series of shell structures surrounding the PTV and an associated set of optimization criteria that carefully guide the optimizer to a solution that complies with the RTOG criterion. What is most original in this work is not the initial five shell structures but the OptiForR50 shell that overlaps the other five shells, the shell expansion margin (M) calculated via Eq. (14) and the carefully calculated optimization criterion (%Vopti) calculated by Eq. (15) that, when met, ensures a plan with a R50% value that complies with the RTOG standard. This methodology translates the R50% “minor violation” lower bound ($LB_{R50\%}^{\mathrm{RTOG}}$) from the RTOG table into a criterion that can be entered in the optimizer.

The optimization is also a well‐balanced set of priorities or penalties that are entered into the optimizer to give appropriate weight to each of the criterion. Notice that the penalties assigned place equal significance to meeting the D2cm criterion and the R50% criterion as to meeting the PTV criterion, as tabulated in Table 1. This is important because in Eclipse and many other RTPS, the prescription is forced to be met regardless of other consequences. So, it is important that the R50% criteria (embedded in the structure of OptiForR50 and %Vopti) carry an equal importance in the optimizer. Furthermore, the PTV dose conformity request in the optimizer (Shell1 maximum dose constraint) is half the weight of the R50% request and the mean dose request in Shell1 is the same priority as the R50%.

A significant part of the success of this method is attributed to the construction of the overlapping shells. The OptiForR50 shell completely overlaps the other shells (Shell1 – Shell5), and thus it allows the planner to request very different constraints of the optimizer. The fact that the OptiForR50 shell is given higher priority than most of the other shells allows the optimizer more freedom to distribute the dose in the most advantageous manner. This methodology is highly successful when the optimization shells overlap an OAR because the OptiForR50 shell is large enough that the dose pushed out of the region where the OAR exists can flare into the portion of OptiForR50 that is far from the OAR. This flaring results from a “conservation of integral dose” hypothesized by Reese et al. if dose is pushed out of one region it must appear in another region.^28^

Another advantage of this method is its flexibility as recommendations change. If the RTOG criteria for R50% are changed the optimization criterion %Vopti, and the OptiForR50 expansion margin (M) will automatically scale with the new standard [just replace $LB_{R50\%}^{\mathrm{RTOG}}$ with the new standard in Eqs. (13) and (15)]. The methodology also discourages the making of physically impossible requests of the optimizer. An impossible request of the optimizer often fails to generate a good plan because the optimization criteria are always an incomplete specification: it is impractical to specify the dose to every voxel. Thus, when an impossible request is made of the optimizer, the optimizer tends to place excessive dose in unexpected places that yields an inferior plan. In the Eclipse optimizer the prescription is a “hard constraint” that will always be satisfied following volumetric normalization regardless of the consequences. Thus, the forcing of the prescription may generate unacceptable plans if unreasonable optimization criteria are used.

It is also important that the optimization criteria are slightly more stringent than is needed. This combined with the carefully crafted %Vopti gives the planner better control of the optimizer. Indeed, if one achieves a plan that satisfies the given criteria that do not mean this is an optimal plan, it only means it is a plan that meets the RTOG criteria. It may be possible to achieve an even better plan by further restricting the optimizer criteria.

This methodology was perfected for an Eclipse optimizer but is based on clear criteria that should be adaptable to any modern VMAT optimizer. This technique would likely work even with a classic multiple static field delivery if the beams were inverse planned, but that would need to be tested. An advantage of VMAT delivery with the methodology presented in this work is that the optimizer has significant degrees of freedom to achieve the requested results. The degrees of freedom available to the optimizer in a classic static field delivery are reduced. Thus, the methodology may be less successful with a classic multiple static field delivery.

It is well suited to implement our multishell replanning methodology in an automated context using Application Programming Interface (API) available in Eclipse ver 11.0 onwards. The Shell2–Shell4 are not actually required if using VMAT delivery. Shell5 is convenient because it directly controls the D2cm. Yet, it is shown in the work of Narayanasamy, Desai et al. on D2cm and R50% that if R50% is met, it is unlikely to violate the D2cm criterion.^29^ Thus with VMAT delivery Shell5 may not be necessary but is still useful. Shell2 – Shell4 provide the planner more control of the dose, but another option would be to use the Eclipse normal tissue objective (NTO) instead.^30^ If the planner is trying to push the plan beyond the initial optimization by this methodology that meets the RTOG criteria, it is best to focus on the OptiForR50 shell and not change criterion on Shell2 – Shell5 or NTO.

Because the development presented here uses the inputs of V_PTV_ and $LB_{R50\%}^{\mathrm{RTOG}}$, this methodology should be applicable to any highly conformal planning system on any body site if the equivalent of $LB_{R50\%}^{\mathrm{RTOG}}$ is specified. This could be an institution’s internally agreed to standard for R50%, or a standard from some other source. Indeed, the work of Hong et al. on spine SBRT used the RTOG 0915 lung criterion for R50% because no other standard was declared for spinal SBRT.^31^ This methodology can be applied equally to liver SBRT or cranial SRS as long as a goal for R50% is specified by a standard setting body, the institution, or even the planner’s own judgment. The methodology is flexible and completely scalable.

It is also interesting to note that as a discipline we use the RTOG 0915 criterion given in Table 1 of the study protocol. This table is the initial study protocol, not the results of the study. The standards in the RTOG provided a standard for the plans in the study based on the best clinical judgment of the study designers, not the analysis of the study results. The results of the RTOG study might be carefully mined to refine the criteria. Until that time, the $LB_{R50\%}^{\mathrm{RTOG}}$ should be considered as a starting point and some effort should be made to do better than the $LB_{R50\%}^{\mathrm{RTOG}}$.

Finally, we note that because all the characteristics of this methodology are defined mathematically, it should be possible to script this procedure and thus create a set of structures and optimization criteria automatically. This could save valuable clinical time and reduce variation between planners.

## 5 CONCLUSIONS

The novel treatment planning methodology proposed here involves a new optimization shell structure (OptiForR50) designed by expansion of the PTV according to Eqs. (8) and (14) and criterion specified in Eq. (15) in addition to more conventional shells structures. This retrospective study of planning methodology showed significant gains in improving target dose conformity, dose falloff outside target, as well as lowering radiation dose to normal lung, heart, and aorta on lung SBRT plans. The methodology also automatically guides the optimizer to generate plans that conform to the RTOG 0915 criteria. Treatment‐related toxicity and tumor control rates need further investigation from the patient follow‐up.

## AUTHOR CONTRIBUTIONS

Dharmin Desai: Conception of study & Proof‐reading manuscript.

Ganesh Narayanasamy: Writing manuscript.

Milan Bimali: Statistics, writing manuscript.s

Ivan Cordrey: Writing & Proof‐reading manuscript.

Hisham Elasmar: Proof‐reading manuscript.

Senthamizhchelvan Srinivasan: Proof‐reading manuscript.

Ellis Lee Johnson: Conception of study.
