# Supplementary material for: Cleaning the dose falloff in lung SBRT plan
Source: J Appl Clin Med Phys. 2020 Dec 7;22(1):100–8. doi: 10.1002/acm2.13113 (PMC7856511; doi:10.1002/acm2.13113)
Supplement: Supplementary file 1 [file ACM2-22-100-s001.docx]

**Cleaning the dose falloff in lung SBRT plan**

Dharmin Desai^1^, Ganesh Narayanasamy^2^, Milan Bimali^3^, Ivan Cordrey^1^, Hisham Elasmar^1^, Senthamizhchelvan Srinivasan^1^, Ellis Lee Johnson^4^

^1^ Memorial Hospital, Chattanooga, TN 37404, USA

^2^ Department of Radiation Oncology, University of Arkansas for Medical Sciences, Little Rock, AR 72205, USA

^3^ Department of Biostatistics, University of Arkansas for Medical Sciences, Little Rock, AR 72205, USA

^4^Department of Radiation Medicine, University of Kentucky, Lexington, KY 40536, USA

**Running Title:** Cleaning up the SBRT dose falloff

**Corresponding Author:**

Ganesh Narayanasamy,

Associate Professor, Department of Radiation Oncology,

University of Arkansas for Medical Sciences,

4301 W Markham St, Slot 771

Little Rock, AR 72205

Email: [nganesh76@hotmail.com](mailto:nganesh76@hotmail.com)

**Author Contributions:**

Dharmin Desai: Conception of study & Proof-reading manuscript

Ganesh Narayanasamy: Writing manuscript

Milan Bimali: Statistics, writing manuscript

Ivan Cordrey: Writing & Proof-reading manuscript

Hisham Elasmar: Proof-reading manuscript

Senthamizhchelvan Srinivasan: Proof-reading manuscript

Ellis Lee Johnson: Conception of study
